# Supplementary material for: Potential transmission chains of variant B.1.1.7 and co-mutations of SARS-CoV-2
Source: Cell Discov. 2021 Jun 15;7:44. doi: 10.1038/s41421-021-00282-1 (PMC8203788; doi:10.1038/s41421-021-00282-1)
Supplement: Supplementary file 1 — Supplementary Information [file 41421_2021_282_MOESM1_ESM.pdf]

Supplementary information

Potential transmission chains of variant B.1.1.7 and co-mutations of SARS-CoV-2

Jingsong Zhang, Yang Zhang, Jun-Yan Kang, Shuiye Chen, Yongqun He, Benhao Han, Mo-Fang Liu, Lina, Lu, Li Li, Zhigang Yi and Luonan Chen

Correspondence: Zhigang Yi (zgyi@fudan.edu.cn); Luonan Chen (lnchen@sibs.ac.cn)

This PDF file includes:

Supplementary Text

Supplementary Figures. S1-7

Supplementary Table S1-7

Supplementary Text : Evolutionary trajectories of potential co-mutations

We also studied the evolutionary trajectories of the potential co-mutations over time in detail. Comparing Supplementary Fig. S4a with the main text Fig. 3a, the 25 nucleotide sites with high mutation rates could cluster into eight potential co-mutation patterns. The frequencies of mutations and potential co-mutations were shown in Supplementary Fig. S6a-h. As expected, the top 4 high-frequency mutations converged into a dominant potential co-mutation pattern (green curve of Supplementary Fig. S6a, main text Fig. 3c-f), and such pattern's lineage represented nearly 100% of the samples since early July, 2020. Notably, the potential co-mutations almost always rose in frequency since their appearances. We could also infer that such pattern's lineage will continually dominate all the samples from COVID-19 patients. Both ORF3a_g25563t and NSP2_c1059t were very high-frequency mutations (both more than 30%, Supplementary Table S4) and the trajectory of the co-occurrences almost overlapped with that of NSP2_c1059t (Supplementary Fig. S6b). This pattern maintained a medium co-occurrence rate on the whole. Supplementary Fig. S6c showed the potential co-mutation pattern of three successive sites (N_28881, N_28882, and N_28883). Unlike the patterns shown in Supplementary Fig. S6a and S6b, the co-occurrence rates of the three successive mutations (Supplementary Fig. S6c) increased first and then decreased slightly. The trajectories of co-occurrence mutations in Supplementary Fig. S6e and S6f shared a very similar trend that increased rapidly first, decreased next, and almost disappeared finally. The co-occurrence rates of Supplementary Fig. S6d first rose, and then gradually dropped to near zero. Interestingly, the potential co-mutation patterns of Supplementary Fig. S6g

and S6h occurred when Supplementary Fig. S6d’s patterns disappeared. Fig. 6i illustrates the residue positions of the above eight potential co-mutation patterns. For these patterns, several patterns occurred within one gene (Supplementary Fig. S6i(c), (h)) or between two adjacent genes (Supplementary Fig. S6i(f)), but most of them spanned distant genes (Supplementary Fig. S6i(a), (b), (d), (g), (e)). The long-range potential co-mutations may imply the folding of RNA spatial structure and the possible interactions between/among these sites.

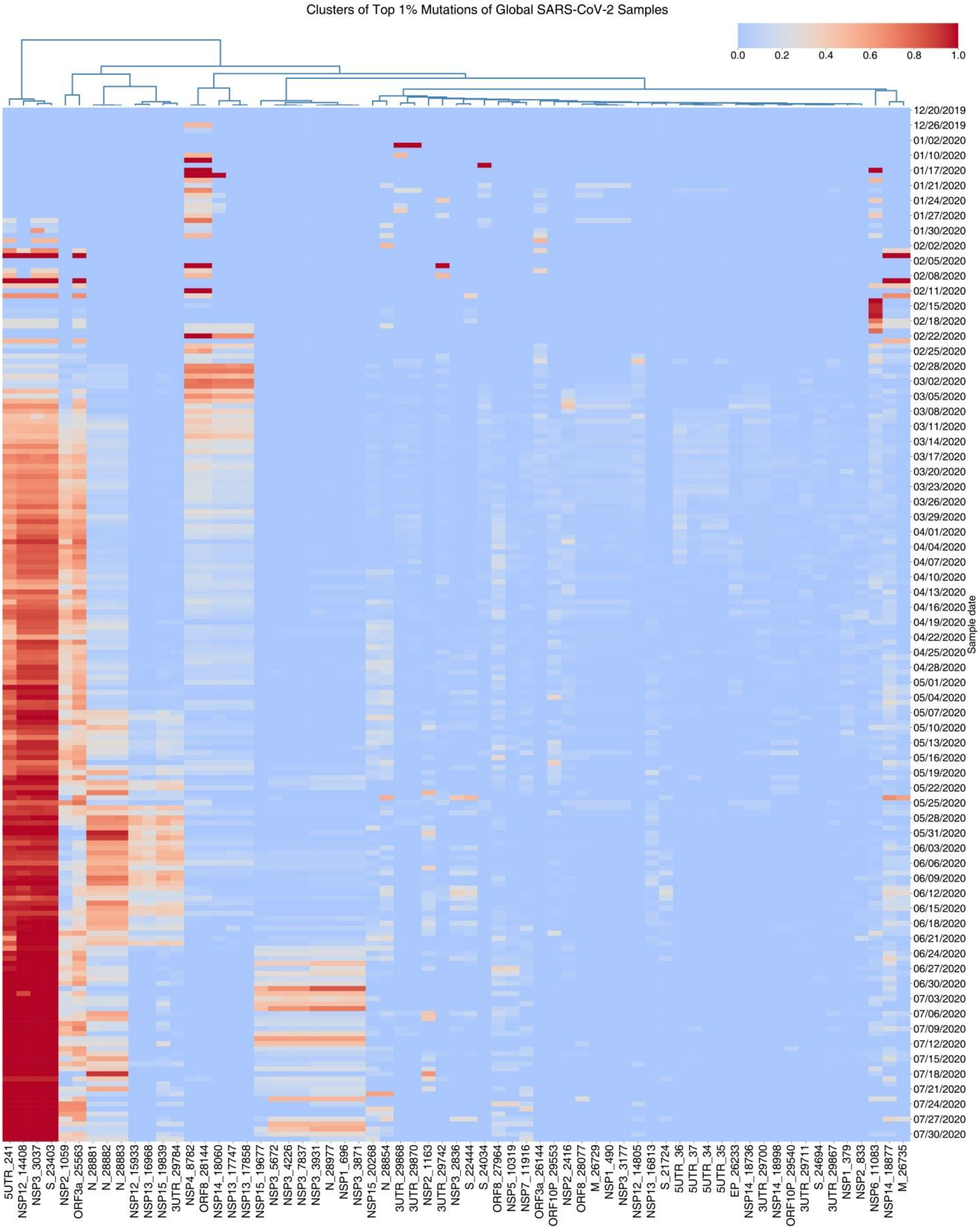

Fig. S1 Ongoing mutations indicate potential co-mutations of SARS-CoV-2 from global samples. The number of sequences per collection date is shown in Supplementary Table S5. Top 1% high-frequency mutations consist of 65 sites. There are about 25 mutations clustered into several potential co-mutation patterns.

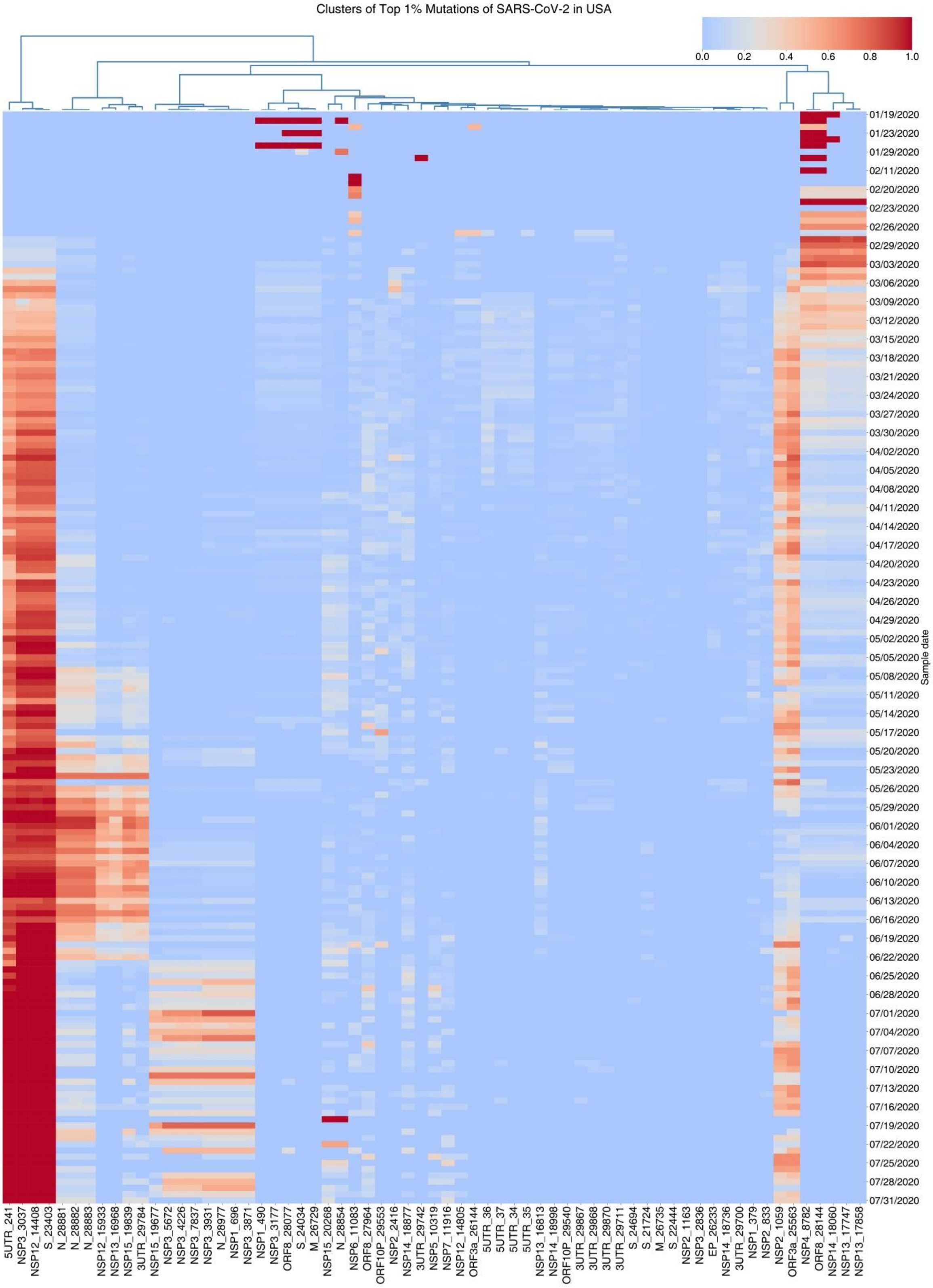

Fig. S2 Ongoing mutations indicate potential co-mutations of SARS-CoV-2 from U.S. samples. The number of sequences per collection date is shown in Supplementary Table S6. Top 1% high-frequency mutations consist of 65 sites.There are also about 25 mutations clustered into several potential co-mutation patterns.

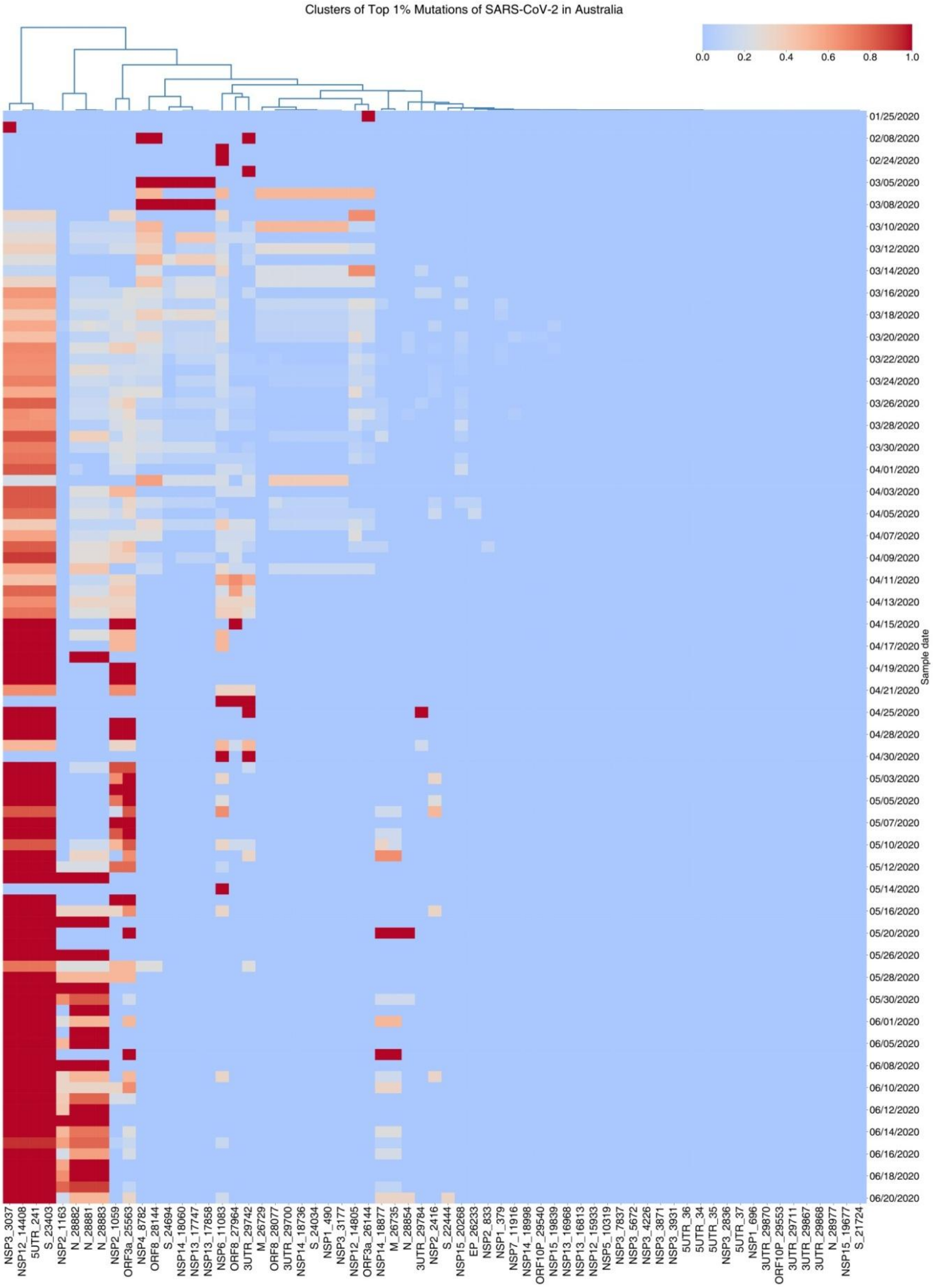

Fig. S3 Ongoing mutations indicate potential co-mutations of SARS-CoV-2 from Australian samples. The number of sequences per collection date is shown in Supplementary Table S7. Top 1% high-frequency mutations consist of 65 sites. There are three possible co-mutation patterns consisting of the top 9 high-frequency mutations.

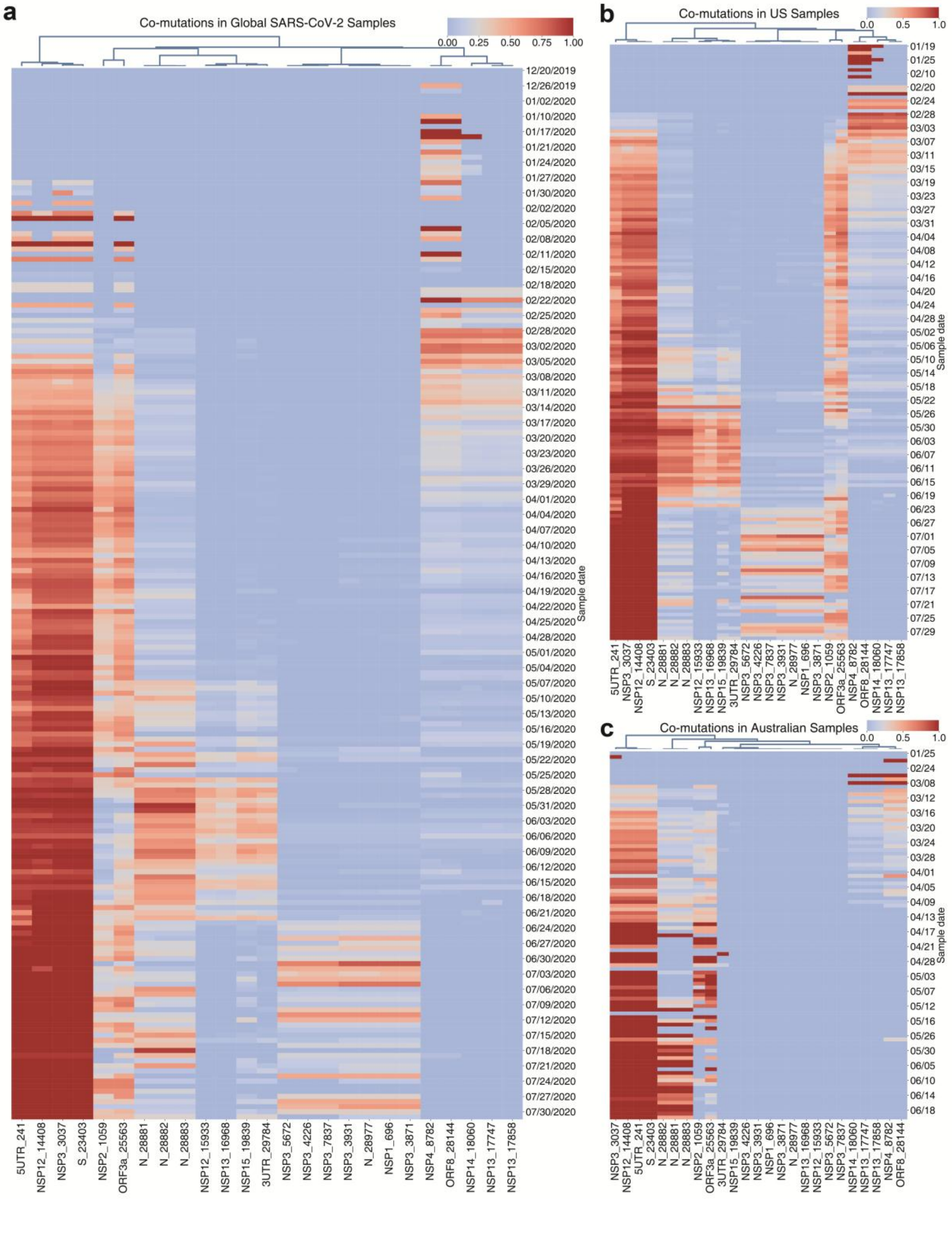

Fig. S4 Ongoing mutations converge into potential co-mutation patterns in SARS-CoV-2 samples. a Landscape of co-mutations of the global SARS-CoV-2 samples. The collection dates of these coronavirus strains range from Dec. 20, 2019 to Jul. 31, 2020. **b**,**c** Landscapes of co-mutations of SARS-CoV-2 samples in USA and in Australia respectively. These 25 sites are almost the top high-frequency mutation sites of SARS-CoV-2. The clusters of the mutations show that several potential co-mutation patterns with different evolutionary trends in not only global samples, but U.S. and Australian ones.

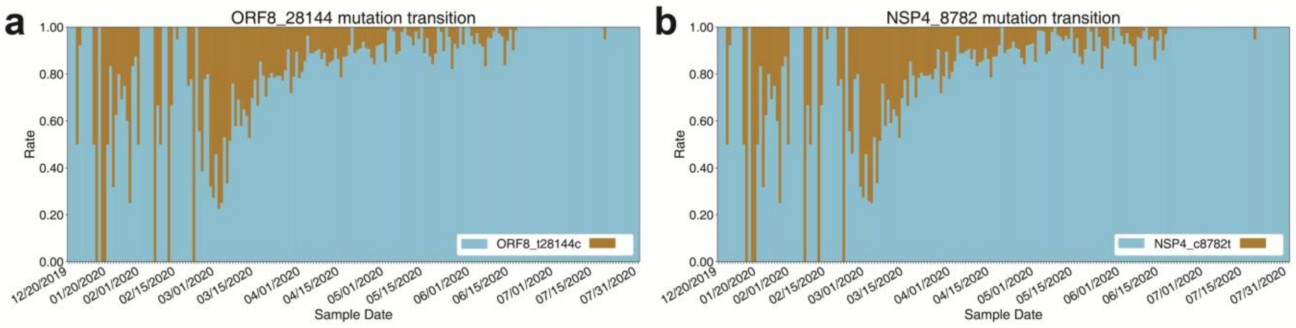

Fig. S5 Mutation transitions of ORF3a_g25563t and NSP2_c1059t. The mutational trends of these two mutations are similar.

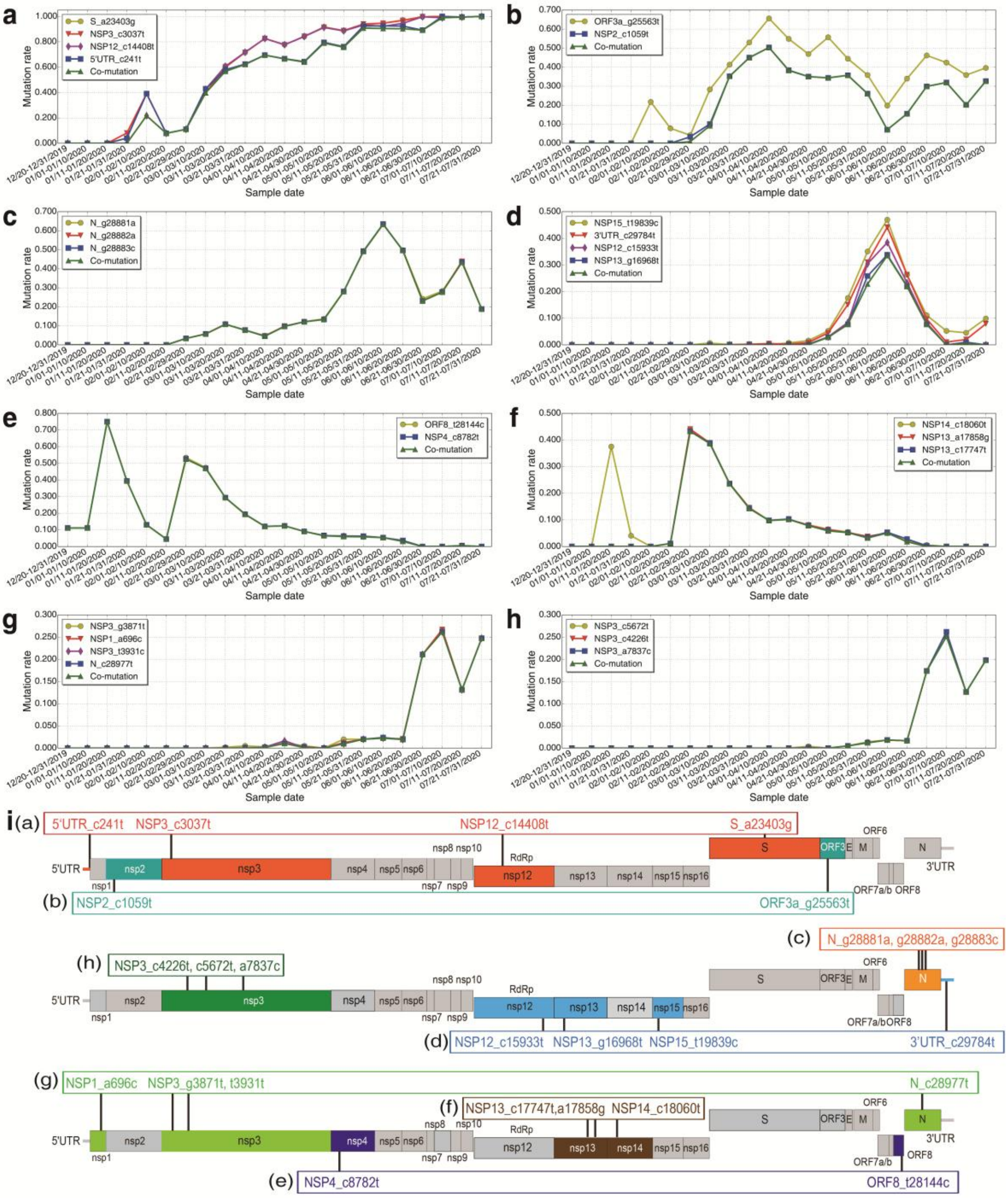

Fig. S6 Evolutionary trajectories of potential co-mutations. a Mutation and co-mutation trends of four sites. Mutations S_a23403g, NSP3_c3037t, NSP12_c14408t, and 5'UTR_c241t all became the dominant variants and such four sites almost co-occur (green curve) over time. **b** The co-mutation rate of ORF3a_g25563t and NSP2_c1059t is nearly the mutation rate of NSP2_c1059t in each time span. **c** The mutations and potential co-mutations of three successive sites almost share an identical trend curve. The co-mutation rates of both **e** and **f** decrease overall, while those of **g** and **h** totally increase. Differently, co-occurrence mutations in **d** appear and increase, then decrease and disappear. **i** The positions of eight potential co-mutation patterns including 25 sites in SARS-CoV-2 genome. Such patterns that reside in multi-genes may imply the potential interactions between/among sites and the evolutionary features.

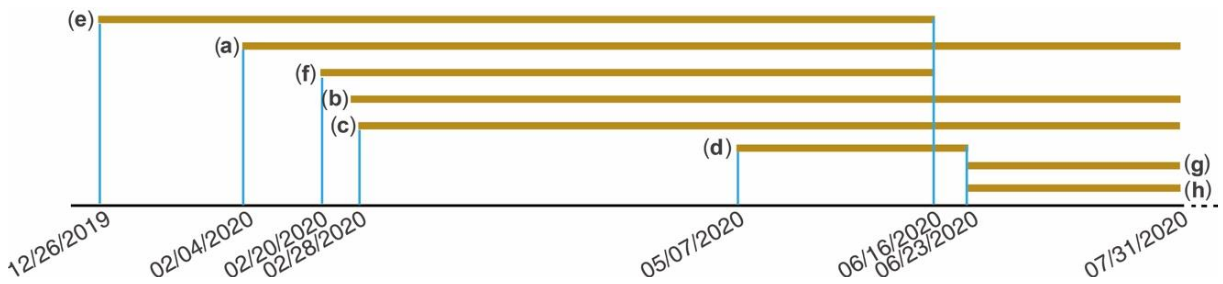

Fig. S7 Highly dynamic co-mutation patterns. The black line at the bottom of the figure is the time axis. Each golden line represents a potential co-mutation pattern that corresponds to Supplementary Fig. S6.

Table S1 Possible animal hosts of the direct progenitor of variant B.1.1.7. The strain labled by a star corresponds with the star variant as shown in main text Fig. 1a.

Strain ID	Host	Collection Location	Collection Date	Mutation
EPI_ISL_752700 (*)	Homo	USA,Hawaii	03/29/2020	D614G,P681H
EPI_ISL_699508	Dog	USA,Texas,Brazos County	07/28/2020	D614G,P681H
MT724346	Tiger	USA	04/04/2020	D614G,T716I
EPI_ISL_641506	Mink	Denmark	08/13/2020	69-70del,D614G

Table S2 Comparative analysis of three possible animal hosts of the direct progenitor of variant B.1.1.7.

Strain ID	Canidae (Dog)	Felidae (Tiger)	Mustelidae (Mink)
Mutation Edit Distance	0	1	2
Spike Similarity	0.0011	0.0015	0.0012
Collection Date	07/28/2020	04/04/2020	08/13/2020
Collection Location	USA	USA	Denmark
#Possible Transmission Origins (Homo)	77	12	45

Table S3 Quantitative analysis of three possible animal hosts based on Supplementary Table S2. The smaller the value, the more similar with the star variant.

Strain ID	Canidae (Dog)	Felidae (Tiger)	Mustelidae(Mink)
Mutation Edit Distance	1	2	3
Spike Similarity	1	3	2
Collection Date	2	1	3
Collection Location	1	1	2
#Possible Transmission Origins (Homo)	1	3	2
Sum	6	10	12

Table S4 Top 1% high-frequency mutations. There are 25 mutations formed eight potential co-mutation patterns. The mutations in the same background color indicate that they co-mutate with each other.

Site	Ref. Site	Mutant	Mut. Rate	Ref. Codon	Mut. Codon	Ref. AA	Mut. AA
S_23403	a	g	0.7764	gat	ggt	D	G
NSP3_3037	c	t	0.7744	ttc	ttt	F	F
NSP12_14408	c	t	0.7720	cta	tta	L	L
5UTR_241	c	t	0.6964	/	/	/	/
ORF3a_25563	g	t	0.4499	cag	cat	Q	H
NSP2_1059	c	t	0.3303	acc	atc	T	I
N_28881	g	a	0.1863	agg	aag	R	K
N_28882	g	a	0.1856	agg	aga	R	R
N_28883	g	c	0.1855	gga	cga	G	R
ORF8_28144	t	c	0.1500	tca	tta	L	S
NSP4_8782	c	t	0.1497	agc	agt	S	S
NSP14_18060	c	t	0.1171	ctc	ctt	L	L
NSP13_17858	a	g	0.1161	tat	tgt	Y	C
NSP13_17747	c	t	0.1160	cct	ctt	P	L
NSP15_19839	t	c	0.0793	ata	aca	I	T
NSP14_18877	c	t	0.0778	gtc	gtt	V	V
3UTR_29784	c	t	0.0723	/	/	/	/
NSP12_15933	c	t	0.0597	acc	atc	T	I
ORF8_27964	c	t	0.0593	gtc	gtt	V	V
NSP6_11083	g	t	0.0544	ttg	ttt	L	F
NSP13_16968	g	t	0.0534	agc	atc	S	I
N_28854	c	t	0.0501	tca	tta	S	L
NSP15_20268	a	g	0.0454	tag	tgg	terminator	G
ORF10P_29553	g	a	0.0416	aag	aaa	K	K
NSP2_2416	c	t	0.0354	tac	tat	Y	Y
5UTR_36	c	t	0.0349	/	/	/	/
M_26735	c	t	0.0283	cag	tag	Q	terminator
5UTR_35	a	t	0.0243	/	/	/	/
5UTR_34	a	t	0.0224	/	/	/	/
NSP3_3871	g	t	0.0224	aag	aat	K	N
NSP2_1163	a	t	0.0217	att	ttt	I	F
NSP12_14805	c	t	0.0216	act	att	T	I
NSP1_696	a	c	0.0205	gac	gcc	D	G
NSP3_3931	t	c	0.0205	gtt	gtc	V	V
N_28977	c	t	0.0204	tct	ttt	S	F
NSP7_11916	c	t	0.0200	tca	tta	S	L
S_22444	c	t	0.0197	gac	gat	D	D
NSP1_379	c	a	0.0188	gtc	gta	V	V
ORF8_28077	g	c	0.0185	cgt	cct	R	P
5UTR_37	c	a	0.0183	/	/	/	/

ORF3a_26144	g	t	0.0183	gtt	ttt	V	F
3UTR_29700	a	g	0.0177	/	/	/	/
NSP3_3177	c	t	0.0174	cct	ctt	P	L
S_24034	c	t	0.0174	aac	aat	N	N
NSP1_490	t	a	0.0169	gat	gaa	D	E
NSP3_2836	c	t	0.0168	tgc	tgt	C	C
NSP3_5672	c	t	0.0168	cct	tct	P	S
NSP3_4226	c	t	0.0167	cca	tca	P	S
NSP3_7837	a	c	0.0167	tta	ttc	L	F
NSP14_18736	t	c	0.0163	gat	gac	D	D
NSP2_833	t	c	0.0161	ttc	ctc	F	L
M_26729	t	c	0.0161	tgt	cgt	C	R
EP_26233	g	t	0.0150	atg	att	M	I
3UTR_29870	c	a	0.0141	/	/	/	/
NSP13_16813	g	a	0.0133	aag	aaa	K	K
NSP15_19677	g	t	0.0132	agg	atg	R	M
NSP5_10319	c	t	0.0126	ctt	ttt	L	F
3UTR_29868	g	a	0.0122	/	/	/	/
3UTR_29711	g	t	0.0120	/	/	/	/
3UTR_29742	g	t	0.0119	/	/	/	/
ORF10P_29540	g	a	0.0111	tgc	tac	C	Y
NSP14_18998	c	t	0.0110	cat	tat	H	Y
S_24694	a	t	0.0104	gga	ggt	G	G
3UTR_29867	t	a	0.0104	/	/	/	/
S_21724	g	t	0.0100	ttg	ttt	L	F

Table S5 Sample numbers of collected dates of SARS-CoV-2 strains. These samples were used for Supplementary Fig. S1 and Supplementary Fig. S4a.

Collection date	Sample number
12/20/2019	1
12/21/2019	1
12/23/2019	1
12/26/2019	2
12/30/2019	13
01/01/2020	2
01/02/2020	2
01/03/2020	1
01/08/2020	2
01/10/2020	2
01/11/2020	1
01/13/2020	1
01/17/2020	1
01/19/2020	3
01/20/2020	2
01/21/2020	6
01/22/2020	22
01/23/2020	8
01/24/2020	5
01/25/2020	13
01/26/2020	8
01/27/2020	5
01/28/2020	8
01/29/2020	12
01/30/2020	8
01/31/2020	4
02/01/2020	2
02/02/2020	2
02/03/2020	3
02/04/2020	1
02/05/2020	3
02/06/2020	1
02/07/2020	3
02/08/2020	2
02/09/2020	1
02/10/2020	5
02/11/2020	1
02/13/2020	3
02/14/2020	1
02/15/2020	19
02/16/2020	21
02/17/2020	32
02/18/2020	8
02/20/2020	4
02/21/2020	9
02/22/2020	3
02/23/2020	2
02/24/2020	9
02/25/2020	13
02/26/2020	9
02/27/2020	15
02/28/2020	25
02/29/2020	33
03/01/2020	24
03/02/2020	31
03/03/2020	24
03/04/2020	32
03/05/2020	54
03/06/2020	33
03/07/2020	54
03/08/2020	45
03/09/2020	55
03/10/2020	83
03/11/2020	80
03/12/2020	116
03/13/2020	229
03/14/2020	155
03/15/2020	147
03/16/2020	167
03/17/2020	157
03/18/2020	165
03/19/2020	213
03/20/2020	226
03/21/2020	174
03/22/2020	130
03/23/2020	207
03/24/2020	241
03/25/2020	242
03/26/2020	185
03/27/2020	202
03/28/2020	178
03/29/2020	95
03/30/2020	162
03/31/2020	194
04/01/2020	231
04/02/2020	214
04/03/2020	191
04/04/2020	133
04/05/2020	99
04/06/2020	167
04/07/2020	128

04/08/2020	124
04/09/2020	73
04/10/2020	66
04/11/2020	60
04/12/2020	49
04/13/2020	111
04/14/2020	88
04/15/2020	70
04/16/2020	87
04/17/2020	73
04/18/2020	52
04/19/2020	25
04/20/2020	54
04/21/2020	83
04/22/2020	122
04/23/2020	97
04/24/2020	79
04/25/2020	32
04/26/2020	53
04/27/2020	108
04/28/2020	114
04/29/2020	148
04/30/2020	160
05/01/2020	122
05/02/2020	70
05/03/2020	66
05/04/2020	110
05/05/2020	95
05/06/2020	78
05/07/2020	48
05/08/2020	24
05/09/2020	29
05/10/2020	24
05/11/2020	51
05/12/2020	54
05/13/2020	58
05/14/2020	57
05/15/2020	36
05/16/2020	21
05/17/2020	8
05/18/2020	38
05/19/2020	53
05/20/2020	27
05/21/2020	48
05/22/2020	29
05/23/2020	24
05/24/2020	24
05/25/2020	28
05/26/2020	98
05/27/2020	110
05/28/2020	37
05/29/2020	53
05/30/2020	26
05/31/2020	22
06/01/2020	79
06/02/2020	123
06/03/2020	80
06/04/2020	42
06/05/2020	96
06/06/2020	24
06/07/2020	47
06/08/2020	124
06/09/2020	59
06/10/2020	39
06/11/2020	74
06/12/2020	74
06/13/2020	43
06/14/2020	34
06/15/2020	110
06/16/2020	81
06/17/2020	67
06/18/2020	114
06/19/2020	61
06/20/2020	63
06/21/2020	23
06/22/2020	61
06/23/2020	47
06/24/2020	42
06/25/2020	31
06/26/2020	40
06/27/2020	27
06/28/2020	27
06/29/2020	30
06/30/2020	23
07/01/2020	6
07/02/2020	15
07/03/2020	16
07/04/2020	9
07/05/2020	11
07/06/2020	39
07/07/2020	29
07/08/2020	17
07/09/2020	23
07/10/2020	26
07/11/2020	5
07/12/2020	11
07/13/2020	12
07/14/2020	32

07/15/2020	66
07/16/2020	15
07/17/2020	10
07/18/2020	14
07/19/2020	19
07/20/2020	14
07/21/2020	16
07/22/2020	12
07/23/2020	4
07/24/2020	9
07/25/2020	3
07/26/2020	5
07/27/2020	15
07/28/2020	6
07/29/2020	9
07/30/2020	14
07/31/2020	8

Table S6 Sample numbers of collected dates of SARS-CoV-2 strains in USA. These samples were used for Supplementary Fig. S2 and Fig. S4b.

Collection date	Sample number
01/19/2020	3
01/21/2020	1
01/22/2020	2
01/23/2020	1
01/25/2020	2
01/28/2020	1
01/29/2020	4
02/06/2020	1
02/10/2020	1
02/11/2020	1
02/17/2020	8
02/18/2020	6
02/20/2020	3
02/21/2020	7
02/22/2020	2
02/23/2020	1
02/24/2020	5
02/25/2020	2
02/26/2020	3
02/27/2020	7
02/28/2020	19
02/29/2020	30
03/01/2020	18
03/02/2020	30
03/03/2020	21
03/04/2020	26
03/05/2020	50
03/06/2020	33
03/07/2020	48
03/08/2020	39
03/09/2020	36
03/10/2020	52
03/11/2020	51
03/12/2020	92
03/13/2020	208
03/14/2020	128
03/15/2020	108
03/16/2020	132
03/17/2020	119
03/18/2020	109
03/19/2020	174
03/20/2020	158
03/21/2020	128
03/22/2020	81
03/23/2020	145
03/24/2020	194
03/25/2020	206
03/26/2020	156
03/27/2020	153
03/28/2020	150
03/29/2020	65
03/30/2020	126
03/31/2020	167
04/01/2020	208
04/02/2020	202
04/03/2020	170
04/04/2020	120
04/05/2020	84
04/06/2020	154
04/07/2020	111
04/08/2020	107
04/09/2020	61
04/10/2020	55
04/11/2020	40
04/12/2020	38
04/13/2020	100
04/14/2020	77
04/15/2020	67
04/16/2020	81
04/17/2020	69
04/18/2020	47
04/19/2020	22
04/20/2020	51
04/21/2020	77
04/22/2020	110
04/23/2020	94
04/24/2020	75
04/25/2020	27
04/26/2020	38
04/27/2020	93
04/28/2020	90
04/29/2020	111
04/30/2020	153
05/01/2020	116
05/02/2020	27
05/03/2020	43
05/04/2020	103
05/05/2020	72
05/06/2020	66
05/07/2020	43
05/08/2020	23

05/09/2020	19
05/10/2020	8
05/11/2020	44
05/12/2020	45
05/13/2020	55
05/14/2020	52
05/15/2020	32
05/16/2020	15
05/17/2020	5
05/18/2020	36
05/19/2020	49
05/20/2020	25
05/21/2020	44
05/22/2020	29
05/23/2020	9
05/24/2020	4
05/25/2020	27
05/26/2020	92
05/27/2020	81
05/28/2020	33
05/29/2020	52
05/30/2020	14
05/31/2020	13
06/01/2020	57
06/02/2020	72
06/03/2020	54
06/04/2020	41
06/05/2020	67
06/06/2020	21
06/07/2020	18
06/08/2020	94
06/09/2020	53
06/10/2020	36
06/11/2020	40
06/12/2020	25
06/13/2020	17
06/14/2020	18
06/15/2020	61
06/16/2020	60
06/17/2020	41
06/18/2020	58
06/19/2020	47
06/20/2020	46

06/21/2020	23
06/22/2020	59
06/23/2020	42
06/24/2020	36
06/25/2020	25
06/26/2020	37
06/27/2020	24
06/28/2020	26
06/29/2020	23
06/30/2020	23
07/01/2020	6
07/02/2020	13
07/03/2020	15
07/04/2020	8
07/05/2020	11
07/06/2020	15
07/07/2020	14
07/08/2020	17
07/09/2020	20
07/10/2020	25
07/11/2020	4
07/12/2020	11
07/13/2020	11
07/14/2020	25
07/15/2020	14
07/16/2020	13
07/17/2020	7
07/18/2020	1
07/19/2020	5
07/20/2020	5
07/21/2020	12
07/22/2020	12
07/23/2020	4
07/24/2020	9
07/25/2020	3
07/26/2020	5
07/27/2020	11
07/28/2020	6
07/29/2020	9
07/30/2020	14
07/31/2020	8

Table S7 Sample numbers of collected dates of SARS-CoV-2 strains in Australia. These samples were used for Supplementary Fig. S3 and Fig. S4c.

Collection date	Sample number
01/25/2020	1
01/30/2020	4
02/08/2020	1
02/21/2020	2
02/24/2020	1
03/03/2020	1
03/05/2020	1
03/07/2020	2
03/08/2020	1
03/09/2020	3
03/10/2020	10
03/11/2020	7
03/12/2020	11
03/13/2020	8
03/14/2020	9
03/15/2020	9
03/16/2020	8
03/17/2020	16
03/18/2020	24
03/19/2020	23
03/20/2020	41
03/21/2020	38
03/22/2020	39
03/23/2020	38
03/24/2020	31
03/25/2020	27
03/26/2020	21
03/27/2020	33
03/28/2020	20
03/29/2020	19
03/30/2020	25
03/31/2020	16
04/01/2020	12
04/02/2020	5
04/03/2020	12
04/04/2020	12
04/05/2020	13
04/06/2020	10
04/07/2020	12
04/08/2020	11
04/09/2020	11
04/10/2020	7
04/11/2020	9
04/12/2020	5
04/13/2020	3
04/14/2020	8
04/15/2020	1
04/16/2020	4
04/17/2020	2
04/18/2020	1
04/19/2020	1
04/20/2020	1
04/21/2020	3
04/22/2020	1
04/25/2020	1
04/27/2020	2
04/28/2020	1
04/29/2020	6
04/30/2020	1
05/02/2020	7
05/03/2020	3
05/04/2020	3
05/05/2020	4
05/06/2020	6
05/07/2020	2
05/09/2020	6
05/10/2020	6
05/11/2020	3
05/12/2020	9
05/13/2020	1
05/14/2020	3
05/15/2020	2
05/16/2020	3
05/19/2020	4
05/20/2020	1
05/23/2020	1
05/26/2020	1
05/27/2020	4
05/28/2020	4
05/29/2020	1
05/30/2020	6
05/31/2020	1
06/01/2020	4
06/03/2020	1
06/05/2020	2
06/06/2020	2
06/08/2020	1
06/09/2020	6
06/10/2020	3
06/11/2020	5
06/12/2020	7

06/13/2020	5
06/14/2020	4
06/15/2020	15
06/16/2020	5
06/17/2020	7
06/18/2020	6
06/19/2020	10
06/20/2020	6
